# Constraint-induced aphasia therapy in post-stroke aphasia rehabilitation: A systematic review and meta-analysis of randomized controlled trials

**DOI:** 10.1371/journal.pone.0183349

**Published:** 2017-08-28

**Authors:** Jiaqi Zhang, Jiadan Yu, Yong Bao, Qing Xie, Yang Xu, Junmei Zhang, Pu Wang

**Affiliations:** 1 Master of Science in Neurological Sciences, Faculty of Medicine, The Chinese University of Hong Kong, Hong Kong, China; 2 School of Rehabilitation Sciences, West China School of Medicine, Sichuan University, Sichuan, China; 3 Ruijin Rehabilitation Hospital of Shanghai Jiao Tong University, Shanghai, China; 4 Department of Rehabilitation Medicine, Ruijin Hospital, School of Medicine, Shanghai Jiao Tong University, Shanghai, China; 5 Department of Physical Education, Wuhan University of Technology, Wuhan, China; Nanjing Normal University, CHINA

## Abstract

**Background:**

Constraint-induced aphasia therapy (CIAT) has been widely used in post-stroke aphasia rehabilitation. An increasing number of clinical controlled trials have investigated the efficacy of the CIAT for the post-stroke aphasia.

**Purpose:**

To systematically review the randomized controlled trials (RCTs) concerning the effect of the CIAT in post-stroke patients with aphasia, and to identify the useful components of CIAT in post-stroke aphasia rehabilitation.

**Methods:**

A computerized database search was performed through five databases (Pubmed, EMbase, Medline, ScienceDirect and Cochrane library). Cochrane handbook domains were used to evaluate the methodological quality of the included RCTs.

**Results:**

Eight RCTs qualified in the inclusion criteria. Inconsistent results were found in comparing the CIAT with conventional therapies without any component from the CIAT based on the results of three RCTs. Five RCTs showed that the CIAT performed equally well as other intensive aphasia therapies, in terms of improving language performance. One RCT showed that therapies embedded with social interaction were likely to enhance the efficacy of the CIAT.

**Conclusion:**

CIAT may be useful for improving chronic post-stroke aphasia, however, limited evidence to support its superiority to other aphasia therapies. Massed practice is likely to be a useful component of CIAT, while the role of “constraint” is needed to be further explored. CIAT embedded with social interaction may gain more benefits.

## Introduction

Aphasia, the acquired language disorder, is a common functional impairment after stroke. Approximately 40–60% of stroke survivors sustain aphasia at chronic stage [1], which is associated with their life dependence, less social participation, poorer rehabilitation outcomes and worsen quality of life [2–5]. Most conventional interventions for aphasia, such as pharmacological managements and rehabilitation programs (e.g. speech language therapy), have been proposed to be beneficial for improving language functions of stroke patients at acute and subacute phases, in addition to the spontaneous recovery [6, 7]. However, the rehabilitative potential of chronic aphasia is still extremely limited, which becomes a big challenge for doctors, speech language therapists and other professionals.

Patients with stroke greatly benefit from specific training programs, owning to the training-induced brain plasticity [8–15]. Various training strategies for stroke rehabilitation have been raised and then applied in real rehabilitative practice, such as mirror neuron system activation [8–10], enriched environment [11–12], non-invasive brain stimulation [13] and constraint-induced therapy (CIT) [14, 15]. Among them, CIT is one of the most widely used strategies that aimed to avoid the “learned nonuse” in patients with stroke. Therefore, it is also known as the “use-dependent learning”, which includes high-intensity repetitive tasks delivered in a relatively short duration [14, 15]. Constraint-induced movement therapy (CIMT), one of the CITs in the field of rehabilitation, focusing on improving the upper extremity motor dysfunctions due to stroke, have substantial benefits on arm motor functions with a long-term effect [15], even for these patients have reached the chronic stage of stroke [16, 17].

To date, CIT has been expanded into cognitive rehabilitation for patients with stroke, including the aphasia rehabilitation [18]. Constraint-induced aphasia therapy (CIAT), which was firstly developed by Pulvermuller F et al in 2001, has been applied in aphasia rehabilitation through a force-used approach [19]. The original protocol of CIAT has three principles: (1) constraint, the patients are strongly encouraged to use verbal communication approaches rather than the non-verbal ways, like gestures; (2) massed practice, the original CIAT protocol includes a total of 10-day interventions delivered 3 hours per day; and (3) shaping, the difficulty of required tasks is gradually enhanced according to patients’ functional performance [19]. Generally, the benefits of CIAT result from expressing the rehabilitation potentials of the lesioned hemisphere to the most extent, by the revision of learned nonuse [19]. Also, it has been proposed that the recovery of aphasia is correlated with the plasticity of neural activities. One of the pathological brain changes of stroke-induced aphasia is the transcallosal disinhibition, it means that, the activities in the lesioned region are down-regulated owning to brain injuries, and then the hyperactive states are noted in collateral regions. Therefore, re-balancing the bilateral activities of hemispheres might contribute to the rehabilitation of aphasia [20]. Brain activities adjustment related to language improvements, which was induced by CIAT, have been found in previous studies. It is likely that CIAT could positively affect the brain activities related to language processes measured by the electroencephalograph (EEG) or the magnetoencephalography (MEG) [21–23], and induces activation in some regions of the brain observed by the functional magnetic resonance image (fMRI) [24, 25]. Those results showed that CIAT was correlated with the positive neuroplasticity in both hemispheres, and the improvements observed by aphasia assessments after the CIAT intervention were associated with the down-regulation of ipsilesional regions (usually left hemisphere) [22, 23] and the up-regulation of contralesional regions (usually right hemisphere) [21, 26].

An increasing number of clinical controlled trials regarding the effects of the CIAT have been published since 2001 [19, 27–32], and some modified protocols of CIAT, like adding every-day communications with family members [33], have been practiced. The participants of CIAT trials were not limited to chronic stroke patients with aphasia nowadays. Some studies have tried to explore the strength of the CIAT for patients at acute phase and subacute phase [27, 31, 34, 35]. However, some controversies were raised about the components in the CIAT. The main controversy is whether the “constraint” and “high-intensity” are useful. Some studies showed that CIAT with less constraint and less intensity still had the similar effect on post-stroke chronic aphasia [36, 37]. A study comparing the intensive and distributed speech therapy gave a conclusion that intensive speech therapy may be not better than distributed speech therapy with the same dosage [38]. Thus, an increasing number of studies started to pay more attention to the difference between the CIAT with other speech therapy programs delivered in the same intensity, and tried to explain which component of the CIAT is useful. Also, studies on modified CIAT (e.g. Intensive language action therapy, ILAT) highlighted the effect from a special session of social interaction in aphasia rehabilitation [28, 39].

Our review was aimed to systematically evaluate the randomized controlled trials (RCTs) published since 2001, and to answer some questions based on currently available evidence: (1) whether CIAT is superior to conventional speech therapies, in terms of improving the language performance and relevant activities of patients with aphasia, at different stages of stroke (examining the effect of combined components of CIAT); (2) whether CIAT is better than other speech therapies delivered in the same intensity in aphasia rehabilitation, and whether CIAT delivered by a distributed way still has favorable effect in aphasia rehabilitation (examining the effect of constraint); (3) whether CIAT embedded with social activities can gain more benefits (examining the effect of social interaction). By critically appraising RCTs of the CIAT, we may identify the useful components of CIAT for successful aphasia rehabilitation.

## Methods

Five computerized databases, including PubMed (via website), EMBASE (via Ovid), the Cochrane library (via Ovid), Medline (via Ovid) and ScienceDirect (via website), were searched for studies published from 2001 up to 18^th^ January 2017. The keywords used for identifying CIAT were: CIAT, use-dependent learning, constraint induced aphasia therapy, constraint induced language therapy, ILAT and intensive language action therapy. The keywords used for identifying stroke included stroke, hemiplegia, hemiparesis, hemiplegic, cerebrovascular accident, CVA. First author read all identifies titles and excluded the obviously irrelevant papers. Papers were included if: (1) RCTs, or the first phase of RCTs with a cross-over design was also included in the present review; (2) patients had a diagnosis of stroke/cerebrovascular accident (CVA); (3) CIAT, modified CIAT, or ILAT [32] was applied in the study, compared with a control group, including different forms of CIAT, other speech therapy programs or the nonintervention control; Papers were excluded if: (1) quasi-RCTs, non-randomized controlled studies, pre-post design studies or case reports; (2) the CIAT group received additional intervention focus on aphasia (e.g. medicine); (3) study did not explore the useful components of CIAT (constraint, massed practice, shaping, social interaction or others). (4) papers were not published in English. The reference list from previous reviews and included RCTs was assessed for relevant records. Two of the authors read through the potentially relevant full-text independently. Disagreements were resolved by consensus discussion between the two assessors.

### Outcome measures

Primary outcomes were the severity of aphasia (e.g. Western aphasia battery—aphasia quotient, WAB-AQ) and language performances (e.g. Aachen aphasia test, AAT), including total scores and subscale scores if applicable. Secondary outcomes included the subjective experience of language performance (e.g. Communicative activity log, CAL), functional communication and any activity related to language functions.

### Data extraction and quality assessment

The quality of RCTs was evaluated based on Cochrane handbook domains [40]: (1) random sequence generation, (2) allocation concealment, (3) blinding of researchers (4) blinding of participants, (5) blinding of outcome assessors, (6) drop-out and exclusion and (7) intention to treat analysis. Two authors assessed each article independently. Disagreements were resolved by consensus discussion with the third author.

### Data analysis

All statistical analyses were performed using RevMan 5.3 (http://ims.cochrane.org/revman). Mean and standard deviations for each outcome were extracted for each group and pooled to obtain mean difference (MD) and 95% confidence intervals (CI). Heterogeneity was examined using I^2^ statistic. Studies with an I^2^ of 25% to 50% were considered to have low heterogeneity, I^2^ of values of 50% to 75%, and ˃ 75% were considered indicative of moderate and high level of heterogeneity, respectively. Fixed-effect models were used to combine studies if I^2^ test was not significant (*P* for heterogeneity<0.1). Otherwise, random effect models were used. *P* < 0.05 was considered indicative of a statistically significant between-group difference. Publication bias was investigated with funnel plots if more than 10 studies were included in the meta-analysis [40]. If there is not available data or varying outcomes used in different studies, a descriptive analysis would be conducted.

## Results

**Fig 1:**
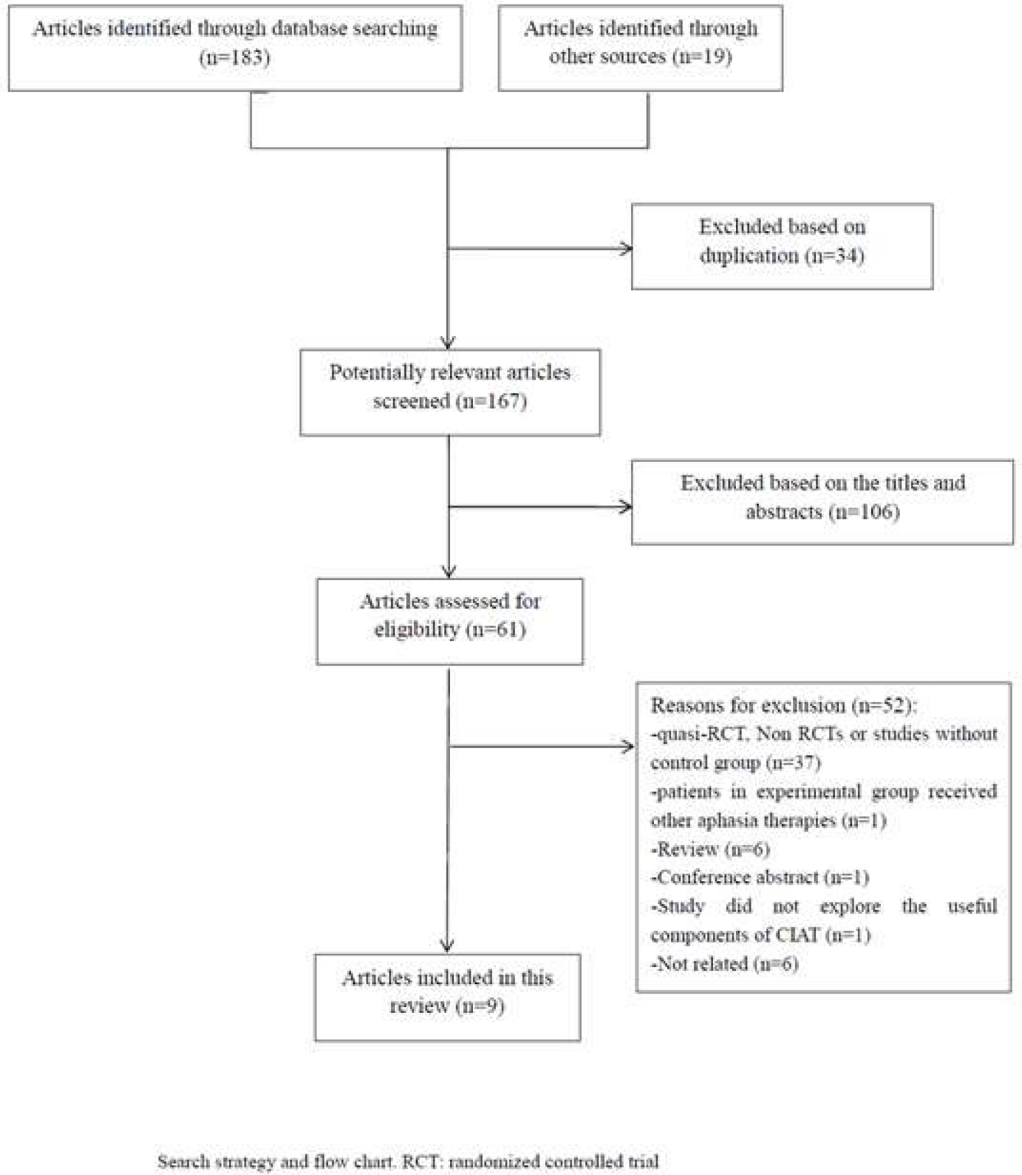
The identification process for selection of trials.

The initial search on computerized databases retrieved a total of 202 citations. After removing duplication, 167 articles were found, of which 57 records were subjected to full-text review. We excluded 48 articles for the following reasons: quasi-RCTs or non-RCTs (n = 7), patients in the experimental arm received other aphasia therapies (n = 1), studies without control groups (n = 34) and reviews (n = 6). One study [41] compared the therapist-led and layperson-led CIAT, so we excluded it. Finally, eight RCTs were included in this review [19, 27–31, 35, 42]. (Fig 1)

Due to various protocols of included studies, we divided them into different groups based on their trial designs: Comparison A: CIAT *vs*. the controls without any component from CIAT; Comparison B: constraint *vs*. unconstraint; Comparison C: social interaction in CIAT, to examine the useful components of the original CIAT protocol. (Tables 1, 2 and 3)

**Table 1:** Characteristics of included studies. Comparison A: CIAT vs. the controls (no any component from CIAT).

First Author Year	Intervention	Participants	Outcomes and Time points	Main results
Pulvermuller F et al 2001 [19]	G1: CIAT (3 hours/day for 10 days) G2: conventional therapy (symptom-specific, same dosage delivered over 4 weeks)	chronic aphasia (one patient in control group was at 2 months after stroke) due to stroke G1/G2 = 10/7 **Type of aphasia:** G1: 6 Broca, 2 Wernicke, 1 Amnesic, 1 Transcortical. G2: 4 Broca, 2 Wernicke, 1 conduction. **Severity of Aphasia (AAT):** G1: 2 mild; 5 moderate and 3 severe G2: 2 mild; 4 moderate and 1 severe	1 AAT (baseline, posttreatment) 2 CAL (baseline, posttreatment)	AAT (G1>G2 in naming, comprehension and token test, but not in repetition test.) CAL (G1>G2)
Szaflarski JP et al 2015 [29]	G1: CIAT (4 hours/day for 10 days) G2: no intervention	chronic aphasia due to stroke G1/G2 = 14/10 **Type of aphasia:** unclear **Severity of Aphasia (token test)** G1: 6 mild; 2 moderate and 6 severe G2: 2 mild;4 moderate and 4 severe	1 BNT; (baseline, posttreatment, 12- week follow-up) 2 Controlled oral word association test (baseline, posttreatment, 12-week follow-up); 3 SFT (baseline, posttreatment, 12-week follow-up) 4 BDAE- complex ideation subtest (baseline, posttreatment, 12-week follow-up) 5 Mini-CAL (baseline, 12-week follow-up)	Mini-CAL (G1>G2, in 12-week follow-up) G1 = G2 in other assessments
Woldag H et al 2016 (both comparison A and B) [27]	G1: CIAT (3 hours/day for 10 days) G2: intensive conventional communication group treatment (3 hours/day for 10 days) G3: control (individual therapy, 14 hours at total in 10 days)	acute phase (mean = 18.7 days after onset) of aphasia due to stroke G1/G2/G3 = 20/20/20 **Type of aphasia** G1: 5 global, 6 Wernicke, 3 Broca, 4 amnesic and 2 others; G2: 4 global, 8 Wernicke, 2 Broca, 5 amnesic and 1 others; G3: 6 global, 7 Wernicke, 3 Broca, 3 amnesic and 1 others. **Severity of Aphasia** unclear	1 AAT (baseline, posttreatment) 2 CAL (baseline, posttreatment)	AAT: G1 = G2 = G3 CAL-quality: G1>G2/G3 CAL-quantity: G1 = G2 = G3

Note: G1 = G2 represented that no significant difference was noted between two groups, *p*≥0.05; G1>G2/G1<G2 represented that the between-group difference was statically significant, *p*<0.05. G: Group; CIAT: Constraint-induced Aphasia Therapy; AAT: Aachen Aphasia Test; CAL: Communication Activity Log; BNT: Boston Naming Test; SFT: Semantic Fluency Test; BDAE: Boston Diagnostic Aphasia Examination.

**Table 2:** Characteristics of included studies. Comparison B: constraint vs. unconstraint.

First Author Year	Intervention	Participants	Outcomes and Time points	Main results
Sickert A et al 2013 [31]	G1: CIAT (2 hours/day for 15 days) G2: conventional therapy (same intensity)	subacute aphasia (1 to 4 months after stroke) due to stroke G1/G2 = 50/50 **Type of aphasia** G1: 11 global, 19 Wernicke, 5 Broca, 12 amnesic and 3 others; G2: 11 global, 19 Wernicke, 8 Broca, and 11 amnesic; **Severity of Aphasia** unclear	1 AAT (baseline, posttreatment, 8-week and one-year follow-up) 2 CAL (baseline, posttreatment, 8-week and one-year follow-up)	AAT (G1 = G2 in posttreatment and follow-up) CAL (G1 = G2 in posttreatment and follow-up)
Wilssens I et al 2015 [30]	G1: CIAT (3 hours/day for 10 days) G2: intensive semantic treatment (comparable intensity in same duration)	chronic aphasia due to stroke G1/G2 = 5/4 **Type of aphasia** G1: 3 Wernicke and 2 transcortical sensory; G2: 3 Wernicke and 1 transcortical sensory **Severity of Aphasia (Token test)** all moderate	1 AAT (baseline, posttreatment) 2 BNT (baseline, posttreatment) 3 PALPA (baseline, posttreatment) 4 ANELT (baseline, posttreatment) 5 CETL (baseline, posttreatment) 6 SAT (baseline, posttreatment)	ANELT (G1 = G2) CETL (G1 = G2) AAT (G1: repetition, naming and written language improved, G2: comprehension improved. Token test G1>G2) BNT (G1>G2) PALPA (G1: 1 of 5 improved; G2: 3 of 4 improved) SAT (G1:1 of 5 improved; G2:0 of 4 improved) Auditory lexical decision and non-word repetition (G1: 2 of 5 improved; G2: 2 of 4 improved) Confident: G2>G1
Ciccone N et al 2015 [35]	G1: CIAT-distributed (45–60 minutes/session, 20 sessions for 5 weeks) G2: impairment-based conventional therapy (45–60 minutes/session, 20 sessions for 5 weeks)	acute aphasia due to stroke (within 10 days of stroke onset) G1/G2 = 12/8 **Type of aphasia** G1:2 anomic, 3 Broca, 2 Wernickes, 2 conduction and 3 global G2:1 anomic, 1 Broca, 1 transcortical motor, 2 Wernickes and 3 global **Severity of Aphasia (WAB-AQ)** G1: 2 mild, 3 moderate and 3 severe; G2: 5 mild, 2 moderate and 5 severe	1 WAB-AQ (baseline, posttreatment, 12 and 26-week follow-up) 2 Discourse analysis (baseline, posttreatment, 12 and 26-week follow-up) 3 SAQoL (baseline, posttreatment, 12 and 26-week follow-up)	G1 = G2 in all outcomes at all time points
Kurland J et al 2016 [42]	G1: ILAT (3 hours/day, 10 days) G2: PACE (3 hours/day, 10 days)	chronic aphasia due to stroke G1/G2 = 12/12 **Type of aphasia** G1: 2 Broca, 1 mixed transcortical, 2 Wernicke, 5 anomic, 1 conduction and 1 transcortical motor G2: 1 grossed Wernicke 1 Wernicke, 3 anomic, 2 transcortical sensory, 1 transcortical motor, 1 Broca, 2 global and 1 optic **Severity of Aphasia (WAB-AQ)** G1: 5 mild-moderate, 5 moderate-severe and 2 severe G1: 5 mild-moderate, 4 moderate-severe and 3 severe	1 BDAE-3 (baseline, posttreatment) 2 BNT (baseline, posttreatment) 3 Cookie in content units (baseline, posttreatment) 4 PICA (baseline, posttreatment)	G1 = G2 in all outcomes

Note: G1 = G2 represented that no significant difference was noted between two groups, *p*≥0.05; G1>G2/G1<G2 represented that the between-group difference was statically significant, *p*<0.05. G: Group; CIAT: Constraint-induced Aphasia Therapy; ILAT: intensive language action therapy; AAT: Aachen Aphasia Test; CAL: Communication Activity Log; BNT: Boston Naming Test; SFT: Semantic Fluency Test; PACE: promoting aphasia communicative effectiveness; BDAE: Boston Diagnostic Aphasia Examination; PALPA: Psycholinguistic Assessment of Language Processing in Aphasia; ANELT: Amsterdam Nijmegen Everyday Language Test; CETL: Communicative Effectiveness Index; SAT: Semantic Association Test; WAB-AQ: Western Aphasia Battery-Aphasia Quotient; SAQoL: The Stroke and Aphasia Quality of Life Scale; PICA: Porch Index of Communicative Ability.

**Table 3:** Characteristics of included studies. Comparison C: social interaction in CIAT.

First Author Year	Intervention	Participants	Outcome	Main results
Stahl B et al 2016 [28]	G1: ILAT (focus on communication and social interaction, 3.5 hours/day, 6 days) and following naming test (focus on naming objects, same intensity) G2: naming test (focus on naming objects, same intensity) and following ILAT (focus on communication and social interaction, 3.5 hours/day, 6 days)	chronic aphasia due to stroke G1/G2 = 9/9 **Type of aphasia** G1: 7 Broca and 2 global G2: 7 Broca and 2 global **Severity of Aphasia (Token Test)** G1: 1 light, 4 moderate and 4 severe G2: 1 light, 4 moderate and 2 severe	AAT (baseline, posttreatment)	AAT: ILAT>naming test, independently when the ILAT was given.

Note: G1 = G2 represented that no significant difference was noted between two groups, *p*≥0.05; G1>G2/G1<G2 represented that the between-group difference was statically significant, *p*<0.05. G: Group; CIAT: Constraint-induced Aphasia Therapy; ILAT: intensive language action therapy; AAT: Aachen Aphasia Test.

### Comparison A: CIAT *vs*. conventional therapy

Three trials included in this review compared the CIAT and conventional speech therapy programs (unconstraint, lower intensity and relatively long duration) or the nonintervention group. Two studies focused on patients at chronic stage [19, 29], while one study [27] enrolled patients at acute phase. The study of Pulvermuller F et al [19] reported that the significant difference in favor of CIAT was noted in naming, expression, comprehension and token test, assessed by AAT, and CAL, in relation to the convention speech therapy. Szaflarski J et al [29] showed an inconsistent result that the immediate effect and long-term effect (12-week follow-up) of the CIAT cannot be found when compared with nonintervention group, except the Mini-CAL (*p* = 0.019). Woldag H et al [27] had three groups: (1) CIAT; (2) intensive conventional treatment (the same intensity with CIAT); and (3) the conventional therapy (14-hour at total over 10 days), which showed that no statistical difference can be noted among three groups in both AAT and CAL (Table 1).

### Comparison B: Constraint *vs*. unconstraint

Four studies [30, 31, 35, 42] and Woldag H et al’s [27] trial compared the CIAT and other intensive speech therapy programs (same intensity as the CIAT but without the component of constraint). Wilssens I et al and Kurland J et al [30, 42] focused on the chronic aphasia, while Ciccone N et al [35] and Sickert A et al [31] explored the CIAT for patient with acute and subacute stroke, respectively. Wilssens I et al [30] only enrolled patients with fluent aphasia. The therapy modality in the control group of Wilssens I et al’s [30] study was the semantic therapy (BOX) and the control group of Kurland’s study received the promoting aphasia communicative effectiveness (PACE) intervention, while the remaining studies did not use specific therapy in the control arms. All control groups allowed patients to use any communication modalities. Ciccone N et al [35] did not find the superiority of distributed CIAT (45–60 minutes/session, 20 sessions for 5 weeks) over the control training with the same dosage for stroke patients at very acute phase. Sickert A et al [31] found both groups showed improvement compared with the baseline, however, no significant difference was noted in any outcomes between two group, which was similar in the study of Kurland et al. Wilssens I et al [30] showed that the CIAT positively affected the language production and phonology, while the group of BOX posed more beneficial effects on the language comprehension and semantics, and Kurland J et al [42] found that the CIAT gained better generalization into the untrained words, compared with the PACE group, in picture naming (23.4% for CIAT and 15.2% for PACE, *p*<0.001) (Table 2).

### Comparison C: Social interaction in CIAT

One study used the ILAT, which was an extend form of CIAT embedding verbal utterances in the context of communication and social interaction, in the experiment [28]. This study compared the ILAT with the naming therapy focusing on speech production with the same intensity (3.5 hours/session for 6 days), and showed a significant effect from the ILAT in AAT, relatively to the naming therapy (Table 3).

### Meta-analysis

Meta-analysis was only conducted among studies that employed same outcomes under the same comparison. After screening the characteristic of included studies, we only pooled the AAT subscores from two studies (Willssen I et al and Sicker A et al) and BNT from two studies (Kurland J et al and Willssens I et al).

#### AAT

**Fig 2:**
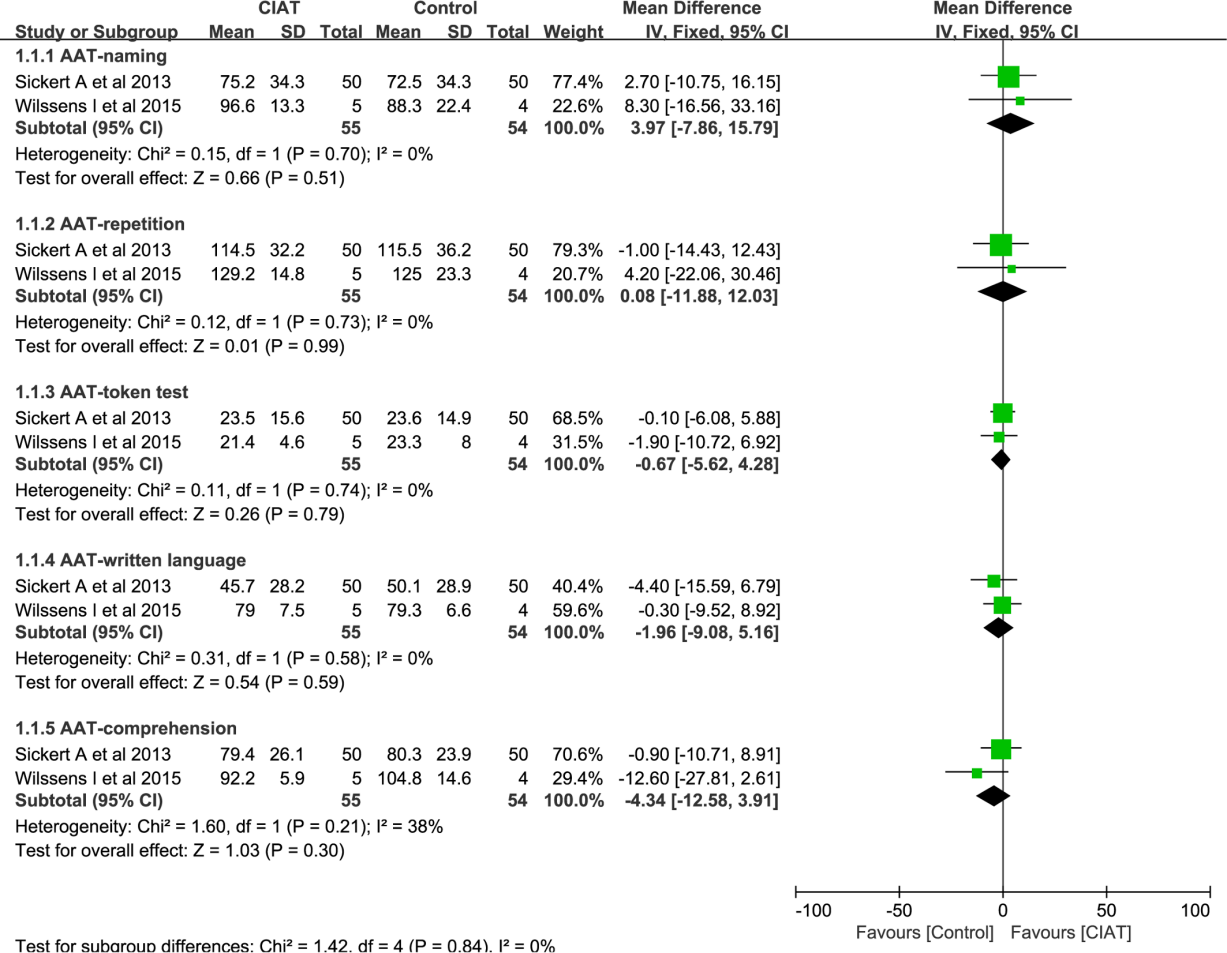
Meta-analysis of AAT subscores.

Available post-treatment data, including naming, repetition, token test written language and comprehension, was recruited in meta-analysis. Meta-analysis revealed that no significant difference was identified between CIAT and other intensive therapy programs, in terms of naming (MD, 3.97, 95% CI, -7.86 to 15.79; *P* = 0.51; I^2^ = 0%), repetition (MD, 0.08, 95% CI, -11.88 to 12.03; *P* = 0.99; I^2^ = 0%), token test (MD, -0.67, 95% CI, -5.62 to 4.28; *P* = 0.79; I^2^ = 0%), written language (MD, -1.96, 95% CI, -9.08 to 5.16; *P* = 0.59; I^2^ = 0%) and comprehension (MD, -4.34, 95% CI, -12.58 to 3.91; *P* = 0.30; I^2^ = 38%). (Fig 2)

#### BNT

**Fig 3:**
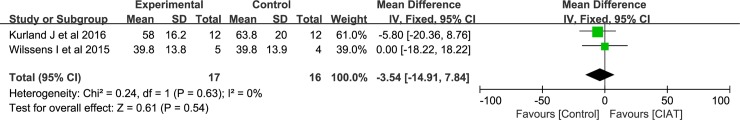
Meta-analysis of BNT.

Meta-analysis revealed that there was no significant difference between the CIAT and other intensive therapy programs, in terms of BNT. (MD, -3.54, 95% CI, -14.91 to 7.84; *P* = 0.54; I^2^ = 0%). (Fig 3)

Therefore, the results of our meta-analyses showed that the CIAT was not superior to other intensive therapy programs.

### Methodological quality assessment

Methodological quality assessment was presented in Table 4.

**Table 4:** Methodological quality of included studies.

Study	Sequence generation	Allocation concealment	Blinding (therapists)	Blinding (patients)	Blinding (Assessors)	Description of losses and exclusion	Intention to treat analysis
Pulvermuller F et al 2001 [19]	Yes	Unclear	No	Yes	Yes	No drop out	NA
Sickert A et al 2013 [31]	Yes	Unclear	No	No	Yes	No drop out	NA
Ciccone N et al 2015 [35]	Yes	Unclear	No	No	Yes	Yes	Yes
Szaflarski JP et al 2015 [29]	Yes	Yes	No	No	Yes	Yes	Yes
Wilssens I et al 2015 [30]	Yes	Yes	No	Unclear	Unclear	No drop out	NA
Stahl B et al 2016 [28]	Yes	Yes	No	Unclear	Yes	No drop out	NA
Woldag H et al 2016 [27]	Yes	Yes	No	Unclear	Yes	Yes	No
Kurland J et al 2016 [42]	Yes	Unclear	No	No	Yes	Yes	No

NA: Not Applicable.

## Discussion

CIAT is likely to be a successful treatment strategy in aphasia rehabilitation, particularly the chronic aphasia, based on currently available evidence. Rather than focusing on activating or modulating the functions of specific brain regions or neuron system, CIAT is mainly aimed to revise the learned nonuse and boost the rehabilitative potential of lesioned hemisphere [19]. Also, the benefits of CIAT can be attributed to training-induced plasticity by balancing the bilateral hemispheres [21–25]. Beside some RCTs included in this review, many case reports or pre-post design studies also showed the benefits of CIAT on chronic aphasia [35, 43–45]. Usually, CIAT was delivered via a therapeutic group. Also, literature provided the evidence about the feasibility of trained laypersons-given CIAT [41], thus, an increasingly number of studies have introduced trained students or volunteers into the therapeutic groups [30, 36], which make it more feasible in rehabilitation settings by saving the human powers. Moreover, recent studies showed that the CIAT implemented at acute phase and subacute phase [27, 31, 35] was tolerable by stroke patients. However, the long-term effects of CIAT were still inconsistent [29, 33].

It seems that the positive effects from CIAT in patients with aphasia, especially in chronic aphasia patients, have been established. Further, we would like to explore whether the CIAT could outperform than conventional aphasia therapy. The first RCT by Pulvermuller F et al [19] provided the evidence that CIAT was superior to the conventional aphasia therapy in improving chronic aphasia patients’ language performance and self-report communication confidence. However, the results were inconsistent with the study of Szaflarski J et al [29], which revealed the CIAT group only could gain more benefits on self-report communication confidence, but not in the language performance, when compared with the nonintervention group. The main problem that hindered us to reach an agreement is the small sample size of these RCTs. Also, literature evidence of the application of CIAT in acute phase of stroke is still limited. Due to the trend of spontaneous recovery, it seems that the advantage of CIAT may not be obvious in comparison with the conventional therapy [27, 31, 35].

The use-dependence neuroplasticity suggests that the high intensity (also called massed practice) is a critical factor in stroke rehabilitation, particularly for chronic stroke patients [14]. A recent systematic review regarding the effect of the high-intensity in aphasia, also supported the opinion that chronic aphasia could be influenced positively by high-intensity speech therapy programs [46, 47]. We could regard “high-intensity (massed practice)” as a useful component of CIAT in aphasia rehabilitation, although the opinion was challenged by a controlled study with small sample, which showed CIAT administered in both intensive and distributed ways with the same dosage resulted in similar positive changes in aphasia severity and language functions [48].

Constraint, the principle focusing on the spoken response and avoiding non-verbal communications, is a controversial point in aphasia rehabilitation. Our studies summarized trials on comparing the CIAT and other intensive speech therapy programs, and did not find the evidence to support the CIAT was superior to the other intensive therapy programs, in terms of improving the language performance [30, 31, 35]. Also, a study compared the CIAT-distributed (constraint and low intensity) and conventional therapy (unconstraint and low intensity), and showed no statistical difference in any outcomes [35]. Some studies with pre-post design reported the effect of less constraint or unconstraint [36, 37]. However, the effect from constraint was found by some study, for example, a case study comparing with constraint and unconstraint therapy, the language performance was in favor of constraint group, which also was associated with the change in fMRI [24]. The principle of constraint in CIAT is changing nowadays, rather than the absolute inhibition of the compensatory communication strategies, researchers tend to allow patients to perform any compensatory strategies that may elicit spoken response [24, 37], thus, real practices become much more difficult, which become a factor that may affect us to explore the real effect of “constraint”. Also, we noted that some benefits were from the constraint, rather than the unconstraint strategies. William I et al [30] showed the CIAT group gained more benefits in the language production, relatively to the BOX group with the same treatment intensity and Kurland J et al [42] mentioned that CIAT may help achieve better generalization to untrained pictures in naming tasks, relatively to the unconstraint treatment with the same high-intensity. It is hard to conclude whether the “constraint” is useful based on RCTs included in this review, however, exploring the special effects from the “constraint” in different aspects of language is still worth in further studies.

Although everyday communication tasks are not available in the original protocol of CIAT, it becomes a common element in further modified CIAT protocols. CIAT plus [33], by adding everyday communications and family involvements in the original CIAT protocol, gained more benefits than the CIAT, in terms of quality of everyday communication. We included one RCT with a cross-over design, which showed that the CIAT embedded with social interaction was superior to the intensive naming therapy, in terms of enhancing patients’ language performance [28]. Social interaction is increasingly paid attention by aphasia rehabilitation practitioners. Lack of generalization of the improvement of language performance in pencil and paper tasks into functional communication is a problem for many previous aphasia studies [1, 49]. In studies of CIAT, rare assessment focused on social participation has been conducted. The only one was CAL, which was used in the first CIAT study [19]. CAL worked as a self-report questionnaire to evaluate the patients’ confidence in communication [19, 29]. Included studies showed that the strength of the CIAT in improving the CAL, in comparison with the control group, which may result from the social interaction [28]. In order to save cost and human power, most CIAT interventions were delivered as the group-based therapy, therefore, the positive effect from the group dynamic cannot be ignored [50]. ILAT, the extend form of CIAT, has paid more attention to the effect of social interaction [28, 39]. Further CIAT studies is needed to regard the functional communication and participation of language-relevant activities as the more important outcomes.

## Limitations

Some limitations could be noted in this review. Firstly, a frim conclusion was unable to be drawn based on the small number of heterogeneous RCTs. Secondly, we can only try to explore the effect from high-intensity (massed practice), constraint and social interaction, however, for other important principles, such as the shaping, could not be well-explained based on currently available literature. Thirdly, we only included the published English language papers. Some unpublished articles, or papers published in other languages might be ignored in this review, which may result in language bias.

## Conclusion

CIAT may be useful for improving chronic post-stroke aphasia, however, limited evidence to support its superiority to other aphasia therapies. Massed practice is likely to be a useful component of the CIAT, while the role of “constraint” is needed to be further explored. Social interaction may be useful for enhancing the benefits of CIAT programs. More rigorous studies about CIAT programs should be conducted before drawing a firm conclusion.

## Supporting information

S1 File Appendix. PRISMA 2009 checklist.
(DOC)

S2 File Appendix. Search strategies.
(DOCX)
