# Supplementary material for: Constraint-induced aphasia therapy in post-stroke aphasia rehabilitation: A systematic review and meta-analysis of randomized controlled trials
Source: PLoS One. 2017 Aug 28;12(8):e0183349. doi: 10.1371/journal.pone.0183349 (PMC5573268; doi:10.1371/journal.pone.0183349)
Supplement: S2 File — Search strategies. (DOCX) [file pone.0183349.s002.docx]

**S2 File. Appendix. Search strategies**

Pubmed: 39 articles

#1 "Hemiplegia"[Mesh]

#2 "Stroke"[Mesh]

#3 CVA

#4 cerebrovascular accident

#5 CIAT

#6 use-dependent learning

#7 constraint induced aphasia therapy

#8 constraint induced language therapy

#9 ILAT

#10 Intensive language action therapy

#11 (#1 OR #2 OR #3 OR #4) AND (#5 OR #6 OR #7 OR #8 OR #9 OR #10)

Cochrane Central Register of Controlled Trials, Embase and MEDLINE through OVID: 58 articles

#1 Hemiplegia

#2 Stroke

#3 CVA

#4 cerebrovascular accident

#5 CIAT

#6 use-dependent learning

#7 constraint induced aphasia therapy

#8 constraint induced language therapy

#9 ILAT

#10 Intensive language action therapy

#11 (#1 OR #2 OR #3 OR #4) AND (#5 OR #6 OR #7 OR #8 OR #9 OR #10)

ScienceDirect: 166 articles

#1 Hemiplegia

#2 Stroke

#3 CVA

#4 cerebrovascular accident

#5 CIAT

#6 use-dependent learning

#7 constraint induced aphasia therapy

#8 constraint induced language therapy

#9 ILAT

#10 Intensive language action therapy

#11 (#1 OR #2 OR #3 OR #4) AND (#5 OR #6 OR #7 OR #8 OR #9 OR #10)
